# The effectiveness of parent training for children with autism spectrum disorder: a systematic review and meta-analyses

**DOI:** 10.1186/s12888-020-02973-7

**Published:** 2020-12-07

**Authors:** Shoumitro (Shoumi) Deb, Ameeta Retzer, Meera Roy, Rupali Acharya, Bharati Limbu, Ashok Roy

**Affiliations:** 1 Imperial College London Faculty of Medicine, London, UK; 2 Centre for Patient Reported Outcomes Research, Institute of Applied Health Research, University of Birmingham, Birmingham, UK; 3 Worcestershire Health and Care NHS Trust, Worcester, UK; 4 Coventry and Warwickshire Partnership NHS Trust, Coventry, UK

**Keywords:** Parent training, Autism, Children, Systematic review, Meta-analysis

## Abstract

**Background:**

Various parent training interventions have been shown to have some effect on the symptoms of children with autism. We carried out a systematic review and meta-analyses to assess effectiveness of parental training for children with autism on their symptoms and parental stress.

**Methods:**

Four electronic databases, CINAHL, EMBASE, MEDLINE and PsycINFO were searched until March 2020 for relevant literature. Two reviewers independently screened bibliographies using an eligibility checklist and extracted data using a structured proforma. We have also carried out meta-analyses when data were available for pooling.

**Results:**

Seventeen papers from 15 studies were included for data analysis. Fifteen papers showed a positive treatment effect when compared with the control group, although not always significant. Meta-analysis based on pooled data from only two studies in each respective intervention, showed small to moderate treatment effects for three interventions, DIR/Floortime, Pivotal Response and Parent focused training respectively.

**Conclusions:**

As in previous systematic reviews there was a mild to moderate treatment effects of three specific types of interventions respectively. However, it was difficult to draw any definitive conclusion about the effectiveness and generalisability of any intervention because of the wide variation in the interventions, control groups, outcome measures, small sample size, small number of studies in meta-analysis, overlap between the intervention and control procedures used in the included studies. There is an urgent need for experts in various international centres to jointly standardise a parent training intervention for children with autism and carry out a large scale RCT to assess its clinical and economic effectiveness.

Research Registry Unique Identifying Number: reviewregistry915.

**Supplementary Information:**

The online version contains supplementary material available at 10.1186/s12888-020-02973-7.

## Background

Autism is a neurodevelopmental disorder, with an estimated prevalence of 0·4% for the core disorder and about 1% for the broad autism spectrum disorder (ASD) [1]. The triad of impairments of social interaction, communication and restricted behaviour patterns have a profound effect on the child’s social development into adulthood and importance of early psychosocial intervention has been advocated in the UK National Autism Plan for Children [2]. The National Institute for Health and Care Excellence, in the UK (Clinical Guideline no. 170) [3] found from meta-analyses that there was small to moderate effects on social interactions, measured by the Autism Diagnostic Observation Schedule (ADOS) [4], joint attention between parent and child, and engagement when caregivers or preschool teachers carried out social communication interventions. Meta-analysis also showed a moderate effect for peer-mediated social communication interventions on peer-child joint engagement for older children (mean ages of 8–9 years) [3]. The guidelines recommend that social-communication programmes may be of help to children with autism, particularly with social isolation. Interpretation of this evidence is difficult due to the variety of comparators and outcome measures used in the trials, as well as the diversity of the interventions included in the clinical effectiveness systematic reviews in terms of the number of intervention sessions, duration of each session and varied components included in different interventions.

A review of parent education programmes for parents of children with ASD examined the formative evaluation of such programmes, including their fidelity to protocols, and their use and reporting of outcomes [5]. Previous reviews of education/training/intervention involving parents of young and school age children with ASD can be considered around specific themes, namely (a) effect on children’s ASD core symptoms, (b) effect on associated symptoms such as challenging behaviour, sleep problems etc., and (c) effect on parental stress, knowledge and confidence in dealing with their children’s behaviour.

### Effect on core ASD symptoms

A Cochrane Review of parent-mediated early intervention for young children with ASD found some evidence for the effectiveness of parent-mediated interventions, particularly in proximal indicators within parent-child interaction, but also in more distal indicators of child language comprehension and reduction in autism severity [6]. This review included studies where interventions were applied to parents and children together to improve their interaction. The authors noted that the ability to draw conclusions from studies would be improved by researchers adopting a common set of outcome measures as the quality of the current evidence is low.

Parson and colleagues [7] conducted a systematic review to examine the existing evidence. Seven studies met the eligibility criteria, including two pre and post cohort studies, three multiple baseline studies, and two RCTs. Interventions included mostly self-guided websites: with and without therapist assistance (*n* = 6), with training videos, written training manuals, and videoconferencing. Preliminary evidence suggested that parent mediated intervention delivered remotely may improve social behaviour and communication skills of ASD children although a high risk of bias existed within all of the studies because of a range of factors including small sample sizes, limited use of standardized outcome measures, and a lack of control groups to negate confounding factors.

Black and Therrien [8] explored the state of research on parental training for school age children with ASD and the value added to these interventions with Parent Training (PT) intervention. The interventions covered social and emotional functioning and problem behaviours. Fifteen PT studies examining 622 child participants with ASD were included and overall, studies demonstrated moderately positive effects for interventions that included PT.

### Effect on associated behaviour

Posterino and colleagues [9] carried out a systematic review and meta-analysis of eight randomised controlled trails (RCTs) on the effect of parent training to manage disruptive behaviour in children with ASD. There were differences in sample size, number of treatment sessions, study duration and control conditions but their results supported efficacy of parent training for disruptive behaviours.

Functional communication training (FCT) involves: 1) identifying the function or purpose of the challenging behaviour; 2) teaching an alternative communicative response; 3) providing function-based reinforcement for the communicative response; and 4) withholding reinforcement following challenging behaviour [10]. Reviews of the literature indicate FCT is an evidence-based practice for children with ASD [11]. Gerow and colleagues [12] conducted systematic descriptive and social validity analyses on 26 peer-reviewed studies on parent-implemented FCT to summarize the extant literature. Across studies, FCT reduced children’s challenging behaviours, and in some cases, the effect was maintained and generalized to new settings. However, few studies reported fidelity data on parent implementation of FCT and data on sustained use by parents. Results also indicated that parents often do not have access to professionals who provide this training in person. Parents living outside urban areas have to rely on interventions delivered remotely.

### Effect on parental outcome

O’Donovan and colleagues [13] found that group-based parent training interventions could modify parent behaviour to achieve improvements in children’s behaviour, skills and socialisation whilst providing social support and coping strategies to address parent health needs. While there was a positive trend for intervention effectiveness, findings were limited by low-quality studies and heterogeneity of intervention content, outcomes and outcome measurement. Training and education empower parents and there are a wide variety of programmes available round the world. Dawson Squibb and colleagues [14] undertook a mixed methods quality appraisal of 32 unique programmes from 20 countries, outside the United States excluding South America. The majority reported positive outcomes but less than one third of the studies met methodological quality criteria.

Our aim was to carry out a systematic review and meta-analysis of published English language studies to evaluate and compare the effectiveness of parent training interventions for parents of children with ASD on children’s ASD core symptoms, associated behaviours such as challenging behaviour and sleep problems, and parental stress, knowledge and confidence in dealing with their children’s behaviour. We found there is not a single type of parent training and different studies used different methods and the components of parent training vary widely among studies. In some studies, the control group also received some intervention usually in the form of information under the umbrella term of ‘psychoeducation.’ As such, the RCTs included were those that were evaluating an enhanced or differing form of parent training. We included studies that provided training directly to parents and excluded studies where children were also involved directly in the intervention procedure.

## Methods

### Search strategy

Our protocol followed the PROSPERO criteria outlined in the PRISMA-P checklist for reporting systematic reviews [15]. Four electronic databases, namely CINAHL, EMBASE, MEDLINE and PsycINFO, were searched for relevant journal articles. Searches on each database were undertaken up to 01.11.2017, with no restrictions on the date of publication. This was subsequently updated using the same search terms and databases up to 01.03.2020. One author (MR) also screened the reference lists of other reviews for eligible articles. Conference abstracts and grey literature were excluded.

The search terms consisted of broad expressions used to describe ASD and support, education and training for parents of children with ASD (see Supplementary material, Appendix A). The search terms were adopted from the systematic reviews carried out to develop a national and an international guide for the use of psychotropic medications for the management of problem behaviour in adults with ID [16, 17] according to the Preferred reporting items for systematic review and meta-analysis protocols (PRISMA-P) [18].

### Study selection criteria

A list of eligibility criteria was adopted from similar reviews on the effectiveness of parent training interventions for children with ASD.

#### Types of studies

Only RCTs that evaluated the effectiveness of parent training interventions for children with ASD were included in this review. The quality of included studies was assessed using the Cochrane Risk of Bias (RoB) checklist [19].

#### Participant characteristics

Participants in the intervention group were all parents of children with ASD, the diagnosis of which was confirmed using a standardised method (clinical diagnosis using DSM/ICD criteria or diagnostic schedules), aged between 1 and 18.

#### Sample size

The minimum sample size was set at ten. There was no upper limit on sample size.

#### Type of intervention

As parent training is an umbrella term that refers to several disparate interventions, we have included studies that included training programmes for parents of children with ASD as the intervention in which the parents were trained by professionals with the ultimate goal of achieving the outcomes described in the next paragraph. Parents must have received ongoing supervision and support from professionals either in person or remotely. The training may have involved group or individual coaching of parents. Additionally, we have included studies in which ‘psychoeducation’ was used as an intervention only when the parent-related outcomes were measured. However, we have excluded studies from meta-analysis in which psychoeducation was used as a control intervention.

##### Outcome measures

Any standardised, measurable, repeatable outcome measures were included. Outcomes included ASD core symptoms such as social interaction, communication and behavioural problems including stereotypy or restricted, repetitive patterns of behaviour, interests or activities. Additionally, non-core ASD symptoms have also been considered in the form of disturbed behaviour or sleep problems. Parent related measures such as parental stress and knowledge have also been included.

##### Meta-analysis

Where data are available on more than one studies using the same intervention, we have pooled data to carry out meta-analyses and produced Forest plots. A random-effects odds ratio model was performed. Heterogeneity was tested using the Chi^2^ test and I^2^ statistic test of heterogeneity and I^2^ > 50% was considered substantial as per the Cochrane guideline [19].

##### Data collection

One author (RA) carried out an initial search using the search strategy, followed by analysis of titles to ensure it has key terms listed. Only published articles were searched. Duplicates and non-human studies were identified and removed manually by two authors (RA and MR). The titles and abstracts retrieved were further analysed against the eligibility criteria independently by two authors (RA and MR). Additional information was sought from two study authors as was necessary to resolve questions about eligibility. The two review authors were blind to each other’s scores whilst using a standardised pre-piloted eligibility criteria checklist. Bibliographies of potential studies were independently screened by RA and MR to identify articles for full text review. The full texts were then reviewed and independently assessed for eligibility by MR and RA using the same eligibility criteria checklist that was used for screening of abstracts and titles. Any disagreement between the reviewers were resolved through discussion or if necessary, through the third author arbitration by SD. In majority of cases, there was agreement between the two reviewers except in two of the papers which were resolved by discussion. The level of agreement was not calculated.

The risk of bias was assessed using Cochrane Risk of Bias tool [19]. Risk of bias tool was applied independently by each reviewer for the paper they reviewed, but interrater reliability was not calculated. Any risks were recorded and disagreements between the review authors over the risk of bias in particular studies were resolved by discussion, with involvement of a third review author (SD) if necessary. However, there was very little disagreement to resolve.

## Results

### Search yield

**Fig. 1:**
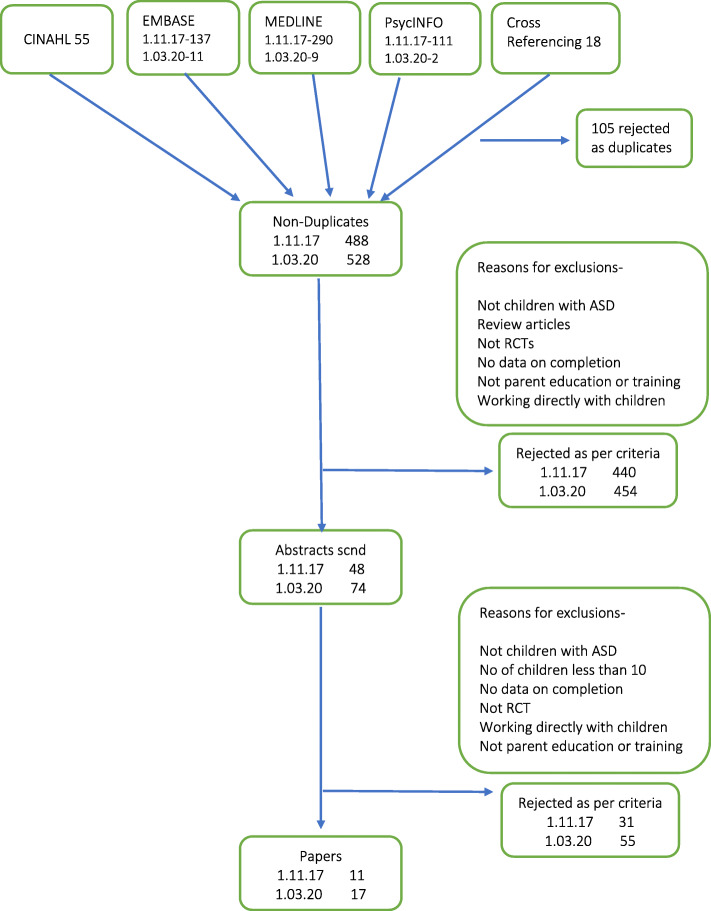
Prisma flow diagram for the literature search process

The initial search identified 528 titles after removing 104 duplicates, and further screening of 528 titles using eligibility criteria yielded 74 abstracts for screening, in two stages, first on 1/11/2017 and then on 01/03/2020. Two of the authors (MR and RA) first independently excluded citations based on inclusion and exclusion criteria. Full texts of the remaining 74 papers were then retrieved and screened independently by two review authors (MR and RA) using the eligibility criteria (see Fig. 1 for initial search findings on 1.11.17, and updated final numbers on 1.03.20). The evidence is listed in Table 1.

**Table 1:** Summary information on included papers

Author (year)	Intervention (*comparator*)	Sample size (*control group size*)	Age (*control group age*)	Males	IQ (*control group IQ*)	N (Follow-up duration)	Outcome measures (Secondary)	Results
Bearss and colleagues (2015)	Parent Training (PT) (*Parent Education Programme) (PEP)*	91 (*89*)	4.7 (1.1) yrs. (*4.8 (1.2) yrs*)	79 *79*	67(73.6%) had IQ 70 or above, 7 or 7.7% had missing IQ, 16 (17.6) had IQ below 70 (*IQ range 67(75.3) had IQ of 70 or above, 13 (14.6) had IQ below 70 while 10 did not have their IQ available)*	16–24 wks (48 wks)	Parent rated ABC-I, Parent rated HSQ -ASD Clinician rated CGI-I VABS	Week 24 PT showed 47.7% decline in Parent ABC-I (from 23.7 to 12.4) compared to 31.8% decrease (23.9 to 16.3%) for PEP (treatment effect, −3.9,95%CI-6.2 to −1.7; *P* < .001, standard effect size =0.62 PT, HSQ declined 55.0% (4.0 at baseline to 1.8 at 24 weeks) compared to a 34.2% decrease (3.8 to 2.5) for PEP (treatment effect, − 0.7; 95%CI, − 1.1 to − 0.3; *P* < .001, standardized effect size = 0.45 On CGI -I, 68.5% (61/89) PT much improved, very much improved compared to 39.6% (36/91) in PEP (*P* < .001); NNT 4(68.5–39.6% = 28.9%;1/28.9 = 3.5 rounded up to 4) For children who showed improvement at week 24, retention was 90% (55/61) at week 48. Of those who did not achieve good response at week 24, 17/21(81%) returned at 48 weeks, mean scores on ABC-I and HSQ-ASD were lower than baseline but showed upward trend from wk24. Available participants *N* = 23 PEP maintained benefit to wk48. On CGI-I, PT, 48/61(79%) remained much improved at 48 weeks; those who did not improve on PT 9/28 (32%) were rated much improved by blind rater. Of the PEP children 16/23 (70%) maintained positive response at 48 weeks.
Iadarola and colleagues 2017	From Bearss and colleagues 2015 Parent training and parent education - Effects on Stress, Strain, and Competence	Same cohort as Bearss and colleagues 2015	Same cohort as Bearss and colleagues 2015	Same cohort as Bearss and colleagues 2015	Same cohort as Bearss and colleagues 2015	24 weeks	PSI PSOC CGSQ Parent rated ABC-I	On the PSI total score, PT showed a 14%reduction, and PEP showed 9.3% reduction. On the CGSQ global score, PT showed 17.2% reduction, and PEP showed 7.1% reduction. For PSOC total score, PT showed 16.4% increase, and PEP showed 7.4% increase. On the PSI difficult child factor, PT produced greater reductions than PEP at week 12 and week 24. The reduction in the PSI total score was greater in PT than PEP at week 24, but the difference was not significant. At week 12 and week 24, PT was superior to PEP on the CGSQ global score and Internalized subscale. The CGSQ Objective subscale reached significance at week 24. On the PSOC, parents in the PT group reported greater gains than parents in PEP at week 12 on the satisfaction subscale but not the efficacy subscale or total score. Improvement was significantly greater in PT compared to PEP on the PSOC total score and the efficacy subscale at week 24. The difference on the satisfaction subscale was no longer significant. Parents in both groups also reported significant decrease in stress (PT: β = − 0.38, *p* = .009; PEP: β = − 0.39, *p* = .006) and strain (PT: β = − 0.50, *p* < .001; PEP: β = − 0.45, *p* < .001) from week 12 to week 24. change in stress and strain from week 12 to 24 did not significantly differ across groups (z = 1.15 and z = 0.74, respectively). Mixed method analysis didn’t show significant difference between groups between 12 and 24 weeks.
Bradshaw and colleagues 2018	From Bearss and colleagues 2015- parental response to active control treatment	Same cohort as Bearss and colleagues 2015	Same cohort as Bearss and colleagues 2015	Same cohort as Bearss and colleagues 2015	Same cohort as Bearss and colleagues 2015	24 weeks.	CGI-S Parent rated-HSQ -ASD, ABC-I, ECI VABS PSI CGSQ PSOC PHQ IQ using Stanford Binet	PEP-R and PEP-NR groups were equally matched were similar on child clinical measures and parent self-report measures, including the CGI-S, ABC, HSQ, ECI, VABS, PSI, CGSQ, or PSOC The only difference between groups was a higher rate of regular educational placement in the PEP-NR group than PEP-R group (60% vs. 36%, *p* = 0.04). The rate of positive response was not different for children with IQ ≥ 70 compared to those < 70. Compared to parents of children in the PEP-NR group, parents of PEP-R children reported significantly greater reductions on the PSI total score, PSI Parent-Child Dysfunctional Interaction subscale, PSI Difficult Child subscale, CGSQ total score, and PHQ-4 total score. Parents of PEP-R also showed greater improvements in the PSOC total score and the PSOC Efficacy domain. There was no significant difference between the children who responded to PEP and PT on ABC, HSQ, VABS or parent reported scales.
Harden and colleagues 2015	Pivotal Response Treatment Group -PRTG (*Psychoeducation – PEG*)	25 (*23*)	4.1 years (1.2) (*4.1 years*) (1.3)	19 *17*	52.8 - DQ *53.5 - DQ*	Final assessment at 12 weeks	SLO CDI VABS MSEL communication subscales	Irrespective of group, children showed significant improvement in total number of utterances across study time points (F(2, 43) =6.12, *p* = .005) Individuals receiving PRTG showed greater improvement (F(2,43) = 3.53,*p* = .038), treatment effect was most apparent for imitative (F(2, 43) = 7.67, *p* = .001) and nonverbally prompted utterances (F(2,43) = 7.06,*p* = .002 Spontaneous utterances showed a nonsignificant trend towards greater improvement in PRTG. At week 12, 21/25 of PRTG and none of PEG met fidelity of PRT implementation. Significant treatment effect observed for Vineland Communication scale, with individuals on PRTG (F(2,19) = 3.80, *p* = .041) Nonsignificant trend for CDI with children with PRTG saying longer utterances. Treatment effect was observed for CGI- severity of social and communication symptoms (F2, 42) =6.84, *p* = .003) and CGI improvement ratings were significantly better in PRTG relative to PEG at weeks 6 and 12 F(1,44) = 15.97, p.001) Older children with higher IQ had more total utterance. Baseline Mullen visual reception scores were a significant predicator of treatment response.
Gengoux, and colleagues 2019	Pivotal Response Treatment -Package-PRT-P *Delayed treatment group-DTG*	23 *(20)*	49.5 (11.2) months *47.2 (10.0)*	21 boys *17 boys*	Not available	Final assessment at 24 weeks	SLO- at baseline, week 12, and week 24 SLO, videos, scored using the BOSCC, higher scores indicate greater impairment The CDI Words and Gestures, The CDI Words and Sentences, VABS MSEL communication subscales CGI-S CGI-I	Children participating in the PRT-P showed significantly greater overall improvement between baseline and week 24 in total number of Utterances (F1,41 = 6.07; *P* = .026) compared with children in the DTG, seen at all time periods, by an increase in nonverbally prompted utterances in PRT-P group (F1,41 = 16.409; P, .001) Improvement in the PRT-P group was observed on the BOSCC social communication subscale and in the BOSCC total score, across the three time points, F2,39 = 17.597; P, 0.001, A significant treatment effect was observed for the CDI words produced out of 396 and CDI words produced out of 680 measures, even when controlling for baseline differences. The treatment effect was also significant on the CGI-S subscale for social communication symptoms (F1,41 = 5.91; *P* = .019). Significant group difference was also evident on the CGI-I subscale (F1,41 = 6.86; *P* < .001). Although not statistically significant, effect-size calculations suggested a medium-size treatment effect for the Vineland-II expressive v-scale score.
Nefdt and colleagues (2010)	Self-Directed learning Program to provide introductory learning in pivotal response treatment -PRT *Wait list Group-WLG*	13 *(14)*	38.92 months (SD = 14.57) *(38.43(SD = 11.20))*	25 males	Not available	No follow up	Fidelity of using PRT procedures; Language opportunities that parents provided; Child’s verbal utterances; Parental confidence – measured on a 6-point scale PSI	Of the 34 dyads who entered the study – 27 (79.4%) completed. Parents in the TG used motivational procedures of PRT F = 107.02 and *p* = .000, effect size 4.12 They provided more language opportunities, F = 91.58 and *p* = .000 and effect size of 2.23 Parent confidence increased, F = 16.37 and *p* = .001, effect size 1.28 There was a significant difference in child utterances, between training and control group F = 16.23 and *p* = .001 and effect size .953 Parents found self-directed learning programme easy to understand, useful and informative, changed the way they interacted with their child and reported that their child was trying to communicate with them
Jocelyn and colleagues (1998)	Autism Preschool Program (*Day care centre with childcare worker*)	16 (*19*)	42 + −9.2 months (*43.8 + − 9.0 months)*	15 *19*	58.4 + −27.5 -Leiter IQ *(67.1 + − 27.5)*	15 weeks	ABC EIDP PDP TRE-ADD Autism Quiz Client satisfaction measure	Mothers and CCW of children in the intervention program reported significant increase in understanding of autism on TRE-ADD Autism Quiz (mothers *p* = 0.02; CCW *p* = 0.008) compared with mothers and CCWs in the control group. Autistic Symptomatology ABC not significantly different. Parents of all children reported improvement over time *p* = 0.000, difference between the two groups was not significant. Developmental outcome – a significant difference was only seen in language score, experimental group language score changed by 5.3 + − 5 months while control group changed by 1.1 + − 4.6 months. Client satisfaction levels – parents reported higher degrees of satisfaction in the experimental group *p* = 0.00007 on knowledge, ability to deal with the needs *p* = 0.009 and how best to meet them *p* = 0.002 compared to parents on the control group.
Malow and colleagues (2014)	Sleep Education – Individualised program (*Group Education Program*)	47 (*33*)	5.6 (2.6) years *5.9 (2.8) years*	39 *25*	27 (64%) IQ > 70 *15 (45%) IQ > 70*	No follow up.	actigraphy -change in latency, wake time after sleep onset, total sleep time CSHQ FISH CBC PSOS RBS-R pedsql	Actigraphy: No difference between the two arms – so results combined which showed improvement in sleep latency, combined mean reduction from 58.2 min to 39.6 with treatment (*p* < 0.0001), in 36 of children, sleep latency was less than 30 min on more than 5 nights per week, there was a modest 2.9% improvement in sleep efficiency and wake time after sleep and total sleep time did not improve. No difference was noted in the questionnaires on how the training was given but in the complete data set, improvements were noted in all the insomnia related parameters, behaviours related to anxiety and depression, withdrawal, attention, repetitive behaviours, parenting efficacy and satisfaction and paediatric quality of life. Parents reported a high level of satisfaction with the program and educator.
Oosterling and colleagues (2010)	Parent based intervention – The Focus Parent Training (*Care as Usual*)	36 (*31*)	24 months (*33.3 months)*	27 25	58 DQ (*58*) DQ	12 months (12 months)	MCDI ADOS CGI-I Erikson Child and Parent Scales	On all language measures there was a main effect of time, meaning that the language skills of children in both groups improved with time. The change in clinical global improvement, as measured with the CGI-I, from baseline to endpoint was not different between the two groups. Regarding engagement, no intervention effects were found. The mothers in the experimental group did not show an improvement in parenting skills relative to the mothers in the control group.
Drew and colleagues (2002)	Social pragmatic joint attention focused parent training programme *(Local Service Model)*	12 *12*	21.4(2.7) months *(23.6(3.8) months)*	11 *8*	88.1(11.2) Nonverbal IQ *(66.0(16.5))*	12 months (12 months)	CDI, Nonverbal IQ- D &E scales of Griffiths Scales of Infant Development. ADI-R PSI	Parent training group had marginally higher language comprehension measured by CDI total words though this missed statistical significance. There were no group differences on words produced or gestures produced. Significantly more children from parent training group moved from nonverbal to having single words or phrase speech (Fisher exact test *p* < 0.05) There was no difference in ADI -R or PSI scores.
Pajareya & Nopmaneejumruslers (2011)	Developmental Individual Difference, Relationship based (DIR) /Floortime™ parent training intervention (*Routine Treatment*)	16 (*16*)	56.6 months (SD 10.1) (*51.5(13.9) SD*)	15 *13*	44.0 (12.9) FEDQ *40.7(15.3) FEDQ*	3 months	FEAS CARS FEDQ	During the study period the intervention group used DIR/Floortime TM at an average of 15.2 h per week SD = 12.4 One family from the intervention group did not complete the study. Analysis including that child showing no improvement, showed that difference in FEAS was significant (F = 4.6, *p* = .045), change in CARS was F = 1.9, *p* = .004, and change in FEDQ was F = 6.4, *p* = .007
Ho and Lin 2020	Training Programme based on the DIR-Developmental Individualised difference Relationship based model *Training Programme based on the developmental milestones.*	12 *(12)*	48.7 (7.4) months *48.3(6.9) months*	All boys *All boys*	Not available	Only post intervention, no follow up.	FEAS CPEP-3 VABS	The FEAS scores for the children and caregivers in the intervention group were much higher than for those in the control group at the end of 14 weeks. The results of repeated measures analyses of variance show that significant interactions were evident between the study group and time for the children’s emotion development, F (1, 22) = 7.559, *p* = .012, η2 = .274, and parenting skills, F (1,22) = 8.447, *p* = .009, η2 = .297. However, no significant interactions were evident between the study group and time for the children’s developmental abilities
Rogers and colleagues (2019)	Early Start Denver Model -ESDM *Treatment as Usual in three sites*	T1 55 T2 51 T3 47 T4 44 *(T1 63* *T2 52* *T3 43* *T4 36*)	T1 20.58(3.37) T2 24.33(3.18) T3 36.47(3.24) T4 48.53(3.05) *T1 20.70(3.21)* *T2 24.13(3.48)* *T3 36.58(3.55)* *T4 48.75(3.77*)	41 *51*	66.98 64.52	24 months (27 months after enrolment)	Language composite age-equivalent score from Expressive Language and Receptive Language age equivalents of the MSEL at each time point. DQ VAB ADOS	When all three sites were taken together, there was a significant change in favour of ESDM but when sites were analysed separately, in sites 1 and 2 there was a significant effect of treatment on the trajectory of language with the ESDM group increasing more than the community group over time. For site 3, although the ESDM group increased, it was less over time than the community group, the group difference was nonsignificant. ADOS and DQ did not differ across groups, no difference in Adaptive behaviour age equivalents.
Sofronoff & Farbotko (2002)	Parent management training aimed to improve parental self-efficacy in management of problem behaviours (*Wait List Control Group*)	69 (*20*)	8 years, 3 months (*8 years, 3 months*)	Not available	-Not available	6 weeks (3 months)	Parental self-efficacy; ECBI Parental assessment of a child’s behaviour problems	Compared with the control group, parents in both intervention groups reported fewer problem behaviours and increased self-efficacy following the interventions, at both 4 weeks and 3 months follow-up. The results also showed a difference in self-efficacy between mothers and fathers, with mothers reporting a significantly greater increase in self-efficacy following intervention than fathers. There was no significant difference between the workshop format and the individual sessions.
Tonge and colleagues (2014)	Parent education and counselling -PEAC; Parent education and behavioural management-PEBM (*Business at Usual*)	70 (*35*)	10 years (*10 years*)	55 32	PEAC group DQ- 48.71 PEBM group −64.74 DQ-63.31	20 weeks (6 months)	VABS DBC PEP RDLS-III	There was a significant improvement in the communication skills of the children whose parents received PEBM compared to the ‘business-as-usual’ control group, but only for the children who had more communication delay. PEBM group performed better than PEAC on VABS – daily living domain, on VABS- socialisation both groups performed better than control group
Keen and colleagues 2010	Professionally supported parent focussed intervention *Self-directed video-based intervention*	17 *(22)*	36.38(7.54) *months* *35.71 (6.92) months*	15 boys *16 boys*	53.06(9.06) *62.86 (6.53)*	3 months	SIB-R CSBS-DP MSEL Parent Measures self-reports PSI PSOC	Both intervention type and parent gender had a significant influence on child-related stress. Fathers experienced higher levels of child-related stress than mothers, the professionally supported intervention reduced child-related stress relative to the self-directed intervention for both mothers and fathers. For self-efficacy there is an interaction for intervention group by baseline score. Parents low in self-efficacy at baseline demonstrated relatively higher levels of self-efficacy if they received the professionally supported intervention than if they received the self-directed intervention.
Tellegen and Sanders 2014	Primary Care Stepping Stones Triple P (PCSSTP). *Care as Usual*	35 *29*	5.66 (2.18) *years* *5.69 (2.2) years*	29 boys *26 boys*	Not available	6 months	ECBI PS DASS 21 PSS Observation of parent child interaction coded according to family observation schedule. PPC RQI	In the short term, there were significant decreases in the intervention group on laxness, F(1, 34) = 26.91, *p* < .001; verbosity, F(1,34) = 22.81, *p* < .001; and over reactivity, F(1, 34) = 30.99, *p* < .001. Parenting confidence found a significant multivariate interaction effect, F (2, 61) = 12.63, *p* < .001, with significant univariate effects on both subscales. A significant multivariate interaction effect on parental adjustment was found, F (4, 59) = 4.56, *p* < .003. Follow-up tests revealed a significant decrease for the intervention group on DASS–21 stress, F(1, 34) = 21.38, *p* < .001, and the PSS, F(1,34) = 24.21, *p* < .001, There was a significant multivariate interaction effect for child behaviour problems, F(2, 61) = 3.33, *p* = .042, with effects on both scales. In the long term, there was maintenance of improvement of child behaviour problems. Dysfunctional parenting styles -scores were still significantly better than preintervention. Differential improvement in parental confidence was significantly maintained. Reduction in stress was significantly maintained. There was no significant change in observed child parent behaviours.

*PT* Parent Training, *PEP* Parent Education Programme, *Parent ABC- I* Parent rated Aberrant Behaviour Checklist -Irritability subscale, *HSQ –ASD* Home Situations Questionnaire-Autism Spectrum Disorder, *CGI-*I Clinical Global Impression–Improvement, *VABS* Vineland Adaptive Behaviour Schedule, *NNT* Number Needed to Treat, *PSI-SF or PS*I Parenting Stress Index-Short Form, *PSOC* Parenting Sense of Competence, *CGSQ* Caregiver Strain Questionnaire, *PEP-R* Parent Education Programme Responders, *PEP-NR* Parent Education Programme Non Responders, *CGI-S* Clinical Global Impression – Severity scale, *ECI* The Early Childhood Inventory, *PHQ* Parent Health Questionnaire, *PRTG* Pivotal Response Treatment Group, *PEG* Psychoeducation, *MB-CDI/CDI* MacArthur Bates Communicative Development Inventories , *MSEL* The Mullen Scales of Early Learning , *PRT-P* Pivotal Response Treatment -Package, *DTG* Delayed Treatment Group, *CGI-I* Clinical Global Impressions Improvement subscales, *SLO* Structured Laboratory observation, *BOSCC* Brief Observation of Social Communication Change, *PRT* Pivotal Response Treatment, *WLG* Wait List Group, *TG* Treatment Group, *CCW* Child Care Workers, *ABC* Autism Behaviour Checklist, *EIDP* Early Intervention Developmental Profile, *PDP* Preschool Developmental Profile, *CSHQ* Children’s Sleep Habits Questionnaire, *FISH* Family Inventory of Sleep Habits, *CBC* Child behaviour Checklist, *RBS-R* Repetitive Behaviour Scale revised, Parent’s proxy report of paediatric quality of life, *ADOS* Autism Diagnostic Observation Schedule, *ADI-R* Autism Diagnostic Interview-Revised, *DIR Intervention* Developmental Individual Difference, Relationship based Intervention, *FEAS* Functional Emotional Assessment Scale, *CARS* Childhood Autism Rating Scale, *FEDQ* Functional Emotional Developmental Questionnaire, *CPEP-3* Chinese version of psychoeducational profile – third edition, *ESDM* Early Start Denver Model, *DQ* Developmental Quotient, *ECBI* Eyberg Child Behaviour Inventory, *PEAC* Parent education and counselling; *PEBM* Parent education and behavioural management, *DBC* Developmental Behaviour Checklist, *PEP* Psychoeducational Profile, *RDLS-III* Reynell Developmental Language Scales III , *SIB-R* Scales of independent behaviour revised, *CSBS-DP* Communication and symbolic behaviour scales developmental profile, *PCSSTP* Primary Care Stepping Stones Triple P, *PS* The Parenting Scale, *DASS 21* Depression, Anxiety, and Stress Scales–21, *PSS* Parental Stress Scale, *PPC* Parent Problem Checklist, *RQI* Relationship quality Index

### Included studies

In total, 17 papers from 15 studies were found from the searches that fulfilled the inclusion criteria for this systematic review. Three publications from the same study were included as different data were presented in these respective papers.

#### Diagnosis of ASD

Different studies used different criteria to confirm the diagnosis of ASD among the children whose parents took part in the study. ADOS [4] and Autism Diagnostic Interview-Revised (ADI-R) [20] along with clinical assessment using Diagnostic and Statistical Manual-IV-Text Review (DSM-IV TR) [21] criteria were most frequently used to establish the ASD diagnosis. Seven studies used ADOS, ADI-R and supporting clinical assessments to establish the diagnosis. Rogers and colleagues [22] used ADOS-Toddler [23] and clinical assessment, while Malow and colleagues [24] used clinical assessments based on DSM-IV criteria [21] along with ADOS [4] to make a diagnosis. Tellegen and Sanders [25] obtained copies of the clinical diagnosis and verified it via a semi structured interview based on DSM-IV-TR [21]. Tonge and colleagues [26] used their own scale, Developmental Behaviour Checklist-Autism Screening Algorithm [27]. Keen and colleagues [28] had a clinical diagnosis based on DSM-IV [21] criteria and ADOS [4], clinical examination. Similarly, three studies used DSM-IV [21] criteria and Jocelyn and colleagues [29] used DSM -III criteria [30]. Ho and Lin [31] used clinical diagnosis based on DSM-V criteria [32].

#### Participant characteristics

##### Age

The studies included parents of children aged from 20.25 months to 10 years. Taking the definition of a toddler as a child up to the age of 36 months, there were four studies where the children were only toddlers [22, 28, 33, 34]. The children in other studies were older but under the age of 10, though toddlers were not excluded.

##### Gender

The 15 studies included 975 children. Two papers reported on different aspects of the same cohort of child parent dyad. One is strictly a parent education study and did not provide information on children’s gender. Of the remaining 886 children from other studies, 743 (83.86%) are boys.

##### Sample size

The number of children included in each study, varies from 12 in each group in two studies [31, 33]; and 91 in Parent Training and 89 in the parent education control group in one study that produced three papers [35–37]. Rogers and colleagues [22] included 118 children at baseline and 80 at the last follow up. Other studies recruited the following numbers-105 [26], 89 [38], 80 [24], 67 [34], 64 [25], 48 [39], 43 [40], 39 [28], 32 [41] and 27 children [42] respectively.

#### IQ level of included children

In view of the wide scatter of developmental stages, different scales have been used to measure level of cognitive functioning of the children. Four studies with younger children [22, 28, 34, 39] and Tonge and colleagues [26] used the Developmental Quotient (DQ) [43].

In Hardan and colleagues’ study [39] both intervention (52.8) and control (53.5) groups were matched for DQ. In Rogers and colleagues’ study [22], the children in the intervention group started with a DQ of 66.98 compared with the control group who had a DQ of 64.52. The children’s DQ in the intervention group was 53.06 and control group 62.86 in Keen and colleagues’ study [28]. In Tonge and colleagues study [26] DQs in two intervention groups were 48.71 and 68.74 respectively, and in the control group 63.31. In the Oosterling and colleagues’ study [34], it was 58. All these values indicated a delayed development in children.

Three papers from one study [35–37], found using an IQ measure that three fourth of the children in both intervention and control group did not have an intellectual disability. Jocelyn and colleagues [29] used Leiter Scale to determine IQ. This is a nonverbal test which is said to be useful for children with autism. The average IQ in the intervention group was 58.4 with a standard deviation (SD) of 27.4, and 67.1 with SD of 27.5 in the control group. Malow and colleagues [24] had 27 (64%) in the intervention group and 15 (45%) in the control group with an IQ above 70. The intergroup IQ difference was not statistically significant. Drew and colleagues [33] using a nonverbal IQ, found an IQ of 88.1 and SD of 11.2 in the intervention and an IQ of 66 and SD, 16.5 in the control group. Pajareya and Nopmaneejumruslers [41] used functional emotional development quotient (FEDQ) [44], the children in the intervention group had FEDQ of 44 while the control group had a mean of 40.7. IQ/ DQ or any level of functioning was not available in five studies [25, 31, 38, 40, 42].

#### Interventions

Of the 17 papers, three [39, 40, 42] used the Pivotal Response Treatment method. Two papers [31, 41] used Developmental, Individualised, Relationship oriented DIR/Floor Time intervention, and another two [33, 34] used parent focussed training. The other studies used a variety of strategies (see Table 2).

**Table 2:** Description of procedures used in intervention and control arms

Intervention arm		Control arm
Language and Communication		
Drew, and colleagues (2002)	The social-pragmatic joint attention focussed parent training programme where speech and language therapists visit parents at home over 6 weeks for 3-h sessions, and demonstrate principles of behaviour management, social pragmatic approach to developing joint attention, nonverbal communication and language skills. The activities for the next 6 weeks were set out in collaboration with the parents, determined by the cognitive and communicative level of the child and their learning style, to be part of play and then to be incorporated into their everyday activities. Therapists were available for telephone support.	Local services – Mixture of speech and language therapy, portage worker input and paramedical input such as occupational therapy and physiotherapy. Three children started 1 to 1 therapy with parents acting as therapists with supervision from Lovaas therapists.
Oosterling, and colleagues (2010)	Focus Parent Training: started with four weekly 2-h sessions with a group of parents, followed by individual 3-h home visits every 6 weeks during the first year. In the second year, the home visits were scheduled at 3-month intervals. The rest of the training was similar to Drew and colleagues as this was replication of the study.	Special day care centres or medical nurseries where on an individual basis, speech and language therapy, motor therapy, music therapy, and play therapy are provided. Psychology input can be arranged from low-frequency sessions with a psychologist (e.g., 1 h per month) to intensive practical support set up in the home environment
Nefdt, and colleagues (2010)	Self-directed learning-Pivotal Response Treatment (PRT): Interactive DVD and accompanying manual covering the procedures used in PRT. DVD was designed to teach parents strategies to increase child motivation to engage in social communication, for providing opportunities for child responses, staying on tasks, and reinforcing attempts, to teach parents basic behavioural techniques such as providing clear prompts and immediate, contingent consequences.	Wait list group
Harden, and colleagues (2015)	Pivotal response treatment group (PRT): Psychologists specializing in PRT utilized the manual How to teach Pivotal behaviours to Children with Autism by Koegel et al. (1989) and a standard set of PRT material and video examples and taught 8, 90 min sessions of parents only consisting of 4 to 6 parents and 1–2 therapists. This was followed by 4 parent child dyad sessions which were individual sessions lasting 60 min with a therapist.	Parent Education Taught by clinical psychology graduate students supervised by a licensed psychologist 12 sessions based on existing autism parent psychology program. 10 sessions parents only groups lasting 90 min. 2 sessions individual parent child dyad sessions with therapist lasting 60 min
Gengoux, and colleagues (2019)	Pivotal Response Treatment Package: Pivotal Response Treatment Package based on a standard set of PRT teaching materials and video examples, Weekly 60-min parent training sessions and 10 h per week of clinician delivered in-home treatment to children from week 1 to 12 followed by monthly 60-min parent training sessions and 5 h per week of in-home treatment for children between weeks 12 and 24	Delayed Treatment Group
Interaction and Play		
Rogers, and colleagues (2019)	Early Start Denver Model 12 weeks - consecutive weeks, sessions with experienced therapists sessions covered a) increasing child’s attention and motivation; (b) using sensory social routines; (c) promoting dyadic engagement and joint activity routines; (d) enhancing nonverbal communication; (e) building imitation skills; (f) facilitating joint attention; (g) promoting speech development; (h) using antecedent-behaviour-consequence relationships (“ABC’s of learning”); (i) employing prompting, shaping, and fading techniques; and (j) conducting functional assessment of behaviour to develop new interventions. Followed by 2 h coaching every 2 weeks. Through enrolment.	Treatment as usual
Pajareya and Nopmaneejumruslers (2011)	Developmental Individual Difference, Relationship based DIR/Floortime™ DIR focusses on the integrated model of human development including interaction with caregivers and the environment, biological, motor and sensory differences, and the child’s functional emotional developmental capacities. Parents attended a one-day training workshop to learn about the model and received a 3-h DVD lecture. This was followed by one on one visits where parents were trained	Routine treatment
Ho and Lin (2020)	Home-based parent-training program based on the DIR Parents received training during the first 2 weeks on DIR, they were provided individualised manuals specific to their children and supported to practice, they were supported at monthly intervals.	Based on the developmental milestones 6 h of training over a three-week period and parent led training not child based.
Behaviour Management		
Bearss, and colleagues (2015) Iadarola, and colleagues (2017) Bradshaw, and colleagues (2018)	Parent Training-11 core sessions 60–90 min, 2 optional sessions, one home visit, over 16 weeks. I home visit and 2 booster phone calls between 16 and 24 weeks, delivered individually.	Parent Education, delivered individually, 12 sessions of 60 to 90 min and 1 home visit over 24 weeks
Tonge, and colleagues (2014)	PEBM skills training. ‘Preschoolers with Autism’ manual-based education and behaviour management skills training package (Brereton and Tonge, 2005). The programme alternates group and individual sessions and focuses on helping parents to discuss their reactions to the diagnosis and to understand more about the problem areas that characterise autism PEAC group. Parents in this treatment only received a manual-based education programme. Emphasis was instead on non-directive interactive discussion and counselling.	Routine treatment.
Malow, and colleagues (2014)	Sleep Study Curriculum covering problems that children with ASD have with sleep, sleep routines, environments etc. Individualised Programme	The same programme but delivered in groups of 2 to 4 parents
Sofronoff and Farbotko (2002)	Parent Training to manage behaviours: Parents attended a workshop which covered 1 psychoeducation 2 comic strip conversations (Gray, 1994a) 3 social stories (Gray, 1994b) 4 management of behaviour problems 5 management of rigid behaviours, routines and special interests 6 anxiety management.	Non-intervention group
Tellegen and Sanders (2014)	Primary Care Stepping Stones Triple P (PCSSTP) PCSSTP is a brief parenting program consisting of four short sessions targeting one or two specific child problems and designed to be accessed through primary health care providers Carried out by individual practitioner to address one or two specific problems. Practitioners had degrees in psychology, they used manuals and adhered to it. Sessions meant to last 15 to 30 min but emphasis on covering content so lasted longer. 4 sessions.	Care as usual group
Parent Education		
Jocelyn, and colleagues 1998	Autism Preschool Program 5 weekly 3 h classes attended by parents and child care workers. Through lectures, videos, and discussion, the following areas were covered – introduction to autism, review of the disorder, behaviour analysis techniques, interventions to encourage and enhance communication, improve social interaction, engage child in play, process of problem solving and program development. Autism Behaviour Specialists visited day care centres 3 h per week for 10 weeks simultaneously to develop goals and approaches although they did not work directly with the child. They worked less intensively with the parents than with the childcare workers.	The control group children attended a day care centre with the support of a childcare worker. The programming was the responsibility of the centre and the community consultants.
Keen, and colleagues 2010	Professionally supported parent focussed intervention The workshop provided information and parent education on the following topics: autism; social; communication; play; sensory; behaviour; strategies to improve social interaction and communication; embedding strategies within daily routines; using a balanced approach; and selecting a child-focused early intervention program. Each topic followed a prescribed format and content that was delivered through a series of power point slides. The following strategies were presented to encourage parental sensitivity and responsivity: following the child’s focus of attention, getting down to the child’s level, augmentative and alternative communication approaches, offering choice, environmental arrangement, imitation and turn taking. Immediately following the workshop, facilitators trained in the assessments and strategies used in the program, made 10 × 1 h home-visits which occurred twice-weekly over 5–6 weeks.	Self-directed video-based intervention, with real life examples about how the strategies could be used to enhance social interaction and communication at home. There were activity sheets modelled on the interactive activities from the DVD that the parents could individualise for their family and incorporate strategies into their daily routines.

The interventions could broadly be divided into those which focussed on improving (a) language and communication, (b) joint attention and play, and (c) behaviours including sleep. In addition, many studies have concentrated on parent education about ASD (Table 2).

##### Language and communication

Drew and colleagues [33] used social pragmatic joint attention focussed parent training programme which was replicated by Oosterling and colleagues [34]. Three papers [39, 40, 42] from two groups, using Pivotal Response Treatment and Rogers and colleagues [22] using Early Start Denver Model, have also focussed on this aspect of autism. All these interventions are categorised under parent mediated language and communication training.

In one pilot study [33] involving 12 children, speech and language therapists visited parents at home and provided focussed training on autism related behaviour management. Language comprehension improved marginally but non-significantly among children in the parent training group compared with the control group. Although there were no intergroup differences in word production, more children in the intervention group moved from being nonverbal to having single words or phrase speech. However, these findings were not replicated in a larger study by Oosterling and colleagues [34] that included 36 children in the intervention group.

Nefdt and colleagues [42] reported on the self-directed learning programme to provide introductory learning in Pivotal Response Treatment training and found significant increase in parents’ confidence (*n* = 13), their ability to provide more language opportunities to their children, and a significant improvement in children’s utterance in the intervention compared with control group. Hardan and colleagues [39] found that although both groups showed significant improvement at follow up, the children whose parents received Pivotal Response Treatment training (*n* = 25) showed a significant improvement compared with those in the control arm in which parents received only psychoeducation (*n* = 23). The same group [40] also found that children participating in the Pivotal Response Treatment programme showed a significantly greater overall improvement between baseline and at week 24 in total number of utterances compared with children in the control group at all time periods.

Rogers and colleagues [22] compared the effects of Early Start Denver Model (ESDM) intervention for children between 12 and 24 months with ASD in three sites with those receiving treatment as usual. One hundred eighteen children were randomised to receive ESDM or treatment as usual and were followed up for 27 months from enrolment, of whom 81 completed treatment. When all three sites were taken together, there was a significant change in favour of ESDM but when sites were analysed separately in sites 1 and 2 there was a significantly better effect of treatment on the trajectory of language development in the ESDM group compared with the control community group. However, in site 3, the intergroup difference was not significant.

##### Interaction and play

Pajareya & Nopmaneejumruslers [41] compared DIR/Floor Time™ intervention with the routine care of preschool children with ASD. They found that after an average of 15.2 h/week of intervention for 3 months, the intervention group made a significantly greater gain in all three outcome measures, namely (a) Functional Emotional Assessment Scale (FEAS) (F = 5.1, *p* = 0.031), (b) Childhood Autism Rating Scale (F = 2.1, *p* = 0.002), and (c) Functional Emotional Questionnaires (F = 6.8, *p* = 0.006).

In an RCT, Ho and Lin [31] compared DIR based parent training with parent education. Parents of 12 children in each group were randomised to receive either a 14-week DIR or parent education. At the end of this period, children in both groups showed improvement in communication. Children in the intervention group showed a significantly greater improvement in functional emotional capacities than those in the control group. Also, the caregivers in the intervention group showed a significantly greater improvement in parenting skills than those in the control group.

##### Behaviour management

Bearss and colleagues [35] evaluated specifically the efficacy of parent training for children with ASD who displayed disruptive behaviour. They carried out a 24-week RCT at six centres comparing parent training (*n* = 89) with parent education (*n* = 91). In the parent training sessions, behaviour strategies were taught to parents which did not happen in the parent education group. The parent training group score improved by 47.7%, on the Aberrant Behaviour Checklist-Irritability (ABC-I) subscale [45] (from 23.7 to 12.4) compared with 31.8% for parent education group (23.9 to 16.3) (*P* < 0.001, standardized effect size = 0.62); the Home Situations Questionnaire-ASD [46] score improved by 55% in the intervention (from 4.0 to 1.8) compared with 34.2% in parent education group (3.8 to 2.5) (*P* < .001, standardized effect size = 0.45); and the positive response for Clinical Global Impression-Improvement (CGI-I) scale [47] were 68.5% for parent training versus 39.6% for parent education (*P* < 0.001).

Tonge and colleagues [26] compared adaptive behaviours in children whose parents had received both education and behaviour management intervention (*n* = 35) with those who had received education and counselling (*n* = 35) and a control group (*n* = 35). Parent education and behaviour management resulted in significant improvement in adaptive behaviour and autism symptoms at 6 months follow-up for children with greater delays in adaptive behaviour.

Malow and colleagues [24] investigated whether sleep education was best provided to parents in an individual or group format to improve sleep and aspects of daytime behaviour and family functioning. Sleep related problems are common in children with ASD. Eighty children, aged between 2 and 10 years with ASD and sleep onset delay took part in the study. Assessments included actigraphy (a non-invasive means of monitoring human rest and activity cycles) and parent questionnaires which were collected at baseline and 1 month after treatment. They found that mode of education, i.e. group versus individual education did not affect the outcomes. Educating parents over a few sessions about sleep, brought about improvement in sleep onset delay as sleep latency, insomnia subscales on the Children’s Sleep Habits Questionnaire, and other outcomes related to child and family functioning improved with treatment.

Sofronoff and Farbotko [38] aimed to help parents of children who were recently diagnosed with high functioning autism. The intervention was compared across two formats, a one-day workshop and six individual sessions, and also with a non-intervention control group. The intervention included psychoeducation combined with the use of comic strip conversations [48] and social stories [49], management of behaviour problems, rigid behaviours, routines and special interests and anxiety. The results indicated that, compared with the control group, parents in both intervention groups reported fewer problem behaviours among children (F = 8.28, *p* < 0.001) and increased self-efficacy (F = 6.26, *p* < 0.001) among parents following the interventions at both 4 weeks and 3 months follow-up. There was no significant difference in outcome between the workshop format and the individual sessions.

Tellegen and Sanders [25] administered Primary Care Stepping Stones Triple P (PCSSTP) to 35 children with ASD and compared with 29 others receiving treatment as usual, in primary care. There was a significant reduction in the short term, in the intensity of behaviour according to the score of both scales used; Eyberg Child Behaviour Inventory (ECBI) [50] (*p* = 0.001), and problem scales, (*p* = 0.004.) and improvement on dysfunctional parenting (*p* = 0.001). These improvements were maintained at 6 months.

##### Education about ASD and effect on parental stress

Jocelyn and colleagues [29] compared in an RCT the effect of psychoeducation for the parents of 16 children with autism with a control group of parents of 19 children who attended day care alone. Mothers and childcare workers of children in the intervention programme reported a significant increase in understanding of autism on TRE-ADD (Treatment Research and Education for Autism and Developmental Disorders) Autism Quiz (Factor et al., Thistletown Regional Centre, Unpublished Data 1987) (mothers: *p* = 0.02; child care workers: *p* = 0.008) compared with mothers and childcare workers in the control group.

Keen and colleagues [28] compared 17 parents of ASD children who had attended a workshop and then received home visits with 22 who had all the information on DVD and found that fathers experienced higher levels of child-related stress than mothers, but the professionally supported intervention reduced child-related stress relative to the self-directed intervention for both mothers and fathers. Parents low in self-efficacy at baseline demonstrated a relatively higher level of self-efficacy if they received the professionally supported intervention than if they received the self-directed intervention.

Iadarola and colleagues [36] provided outcome data on (a) Parenting Stress Index-Short Form (PSI) [51] (b) Caregiver Strain Questionnaire (CGSQ) [52] and (c) Parenting Sense of Competence (PSOC) [53] on the same cohort that was included in Bearss and colleagues’ (2015) study [35]. Parents in Parent Treatment (PT) group reported greater improvement than Parent Education Programme (PEP) group on the PSOC [53] (effect Size = 0.34), CGSQ [52] (effect Size = 0.50), and difficult child subdomain of the PSI [51] (effect Size = 0.44). Parents in both groups reported significant decrease in stress (PT: β = − 0.38, *p* = .009; PE: β = − 0.39, *p* = .006) and strain (PT: β = − 0.50, *p* < .001; PEP: β = − 0.45, *p* < .001) from week 12 to week 24. Bradshaw and colleagues [37] showed that parents in PEP-R (Parent Education Programme-Responders) reported significant reductions on the Parenting Stress Index [51], Caregiver Strain Questionnaire [52], and Parent Health Questionnaire [54], and increases on the Parenting Sense of Competence+ Scale score [53].

#### Design

As per our search criteria, all 15 included studies are RCTs. Tonge and colleagues [26] describe their study as randomized group comparison-the children in the active intervention arms were allocated randomly while selected metropolitan and rural control regions provided 35 families as the control group. Drew and colleagues [33] who used social-pragmatic joint attention focused parent training programme as intervention and Pajareya and Nopmaneejumruslers [32] who used DIR/Floor Time™ intervention, describe their respective studies as pilot.

Sofronoff and Farbotko [38] used parents of children in the waiting list and Keen and colleagues [28] parents who used self-directed help as the control group respectively.

There was a heterogeneity of control groups; parents received psychoeducation in three studies [31, 35, 39]. In Keen and colleagues’ study [28] parents in the control group received training material in the form of a DVD with real life examples about how the strategies could be used to enhance social interaction and communication at home. The parents in the control group in Malow and colleagues’ study [24] received their training in a group, instead of having individual training. In Jocelyn and colleagues’ study [29] the children in the control group attended day care centre with childcare workers. Two studies [40, 42] had wait list and delayed treatment group as the control group. Table 2 sets out the interventions and controls in all the studies.

#### Follow up

The longest follow up was in Rogers and colleagues [22] study (27 months from enrolment). Two studies [33, 34] followed up children for 12 months. One study followed up children for 48 weeks [35], two [25, 26] for 6 months, two [35, 37] for 24 weeks, one [29] for 15 weeks, and three [28, 32, 38] for 3 months respectively. Ho and Lin [31] completed the RCT in 14 weeks, and there was no follow up, this was the case with Hardan and colleagues [39] too who did so in 12 weeks. Two studies [24, 42] did not provide any follow up data.

#### Fidelity

In Bearss and colleagues’ [35] study, postgraduate-educated therapists implemented both Parent Training and Parent Education interventions according to the treatment manuals, only after they had undertaken systematic training and certification. The parent training manual contained verbatim scripts and instructions for therapists. Each site had weekly supervision for therapists and every month, there were teleconferences across the sites to ensure integrity of study interventions. A checklist was used to specify the required elements of each session and independent raters scored treatment integrity on a 10% sample of randomly selected, video-recorded parent training and parent education sessions. Parent training was provided by postgraduate-educated clinicians who were supervised by the first author in Gengoux and colleagues’ [40] study. In Hardan and colleagues’ study [39], parent training was carried out by psychologists specializing in it and parent education group was conducted by psychology graduate students under supervision of a licensed psychologist.

In Malow and colleagues’ study [24], before they undertook interventions, all educators received training in both sleep interventions and had to achieve fidelity criteria. To ensure that the sleep curriculum was being followed across all the sites, the training sessions were video recorded. A single central rater reviewed 73% of the sessions for fidelity. The fidelity criteria used to score the sessions were a) session integrity, b) adherence to the manual, c) characteristics of the educator and d) educator interaction with parents. These were achieved in all the sessions.

The Early Start Denver Model (ESDM) sessions were conducted in the three university clinics by highly experienced and credentialed therapists trained to fidelity by the authors (Rogers and colleagues [22]) who monitored it quarterly throughout the study. Therapist fidelity average score in coaching interactions with the parent was a mean of 3.62 (s.d. =.25), measured on 13 items with scores ranging from 1 to 4.

In Tellegen and Sanders’ study [25], practitioners were a psychology degree holder, were accredited in Primary Care Stepping Stones Triple P (PCSSTP), attended supervision sessions, used a manual and protocol adherence checklist. To assess for protocol adherence, an independent researcher (postgraduate psychology student) familiar with PCSSTP completed adherence checklists while viewing a random sample of 20% of session recordings.

Tonge and colleagues [26] sustained treatment by training therapists in each condition, requiring them to follow a manual that delineated the treatment step by step, videotaping 10% sample of group therapy for content and treatment adherence and therapists receiving clinical supervision and training throughout the study.

Apart from the studies mentioned in this section, no other study provided treatment fidelity data.

#### Meta-analyses

**Fig. 2:**
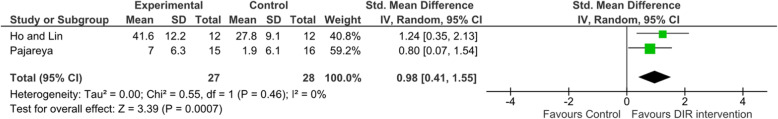
Developmental, Individual Difference, Relationship-based (DIR)/Floortime™ Forest Plot

**Fig. 3:**
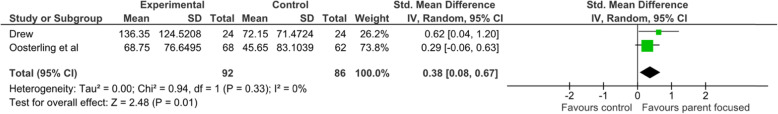
Parent Focussed Training Forest Plot

**Fig. 4:**
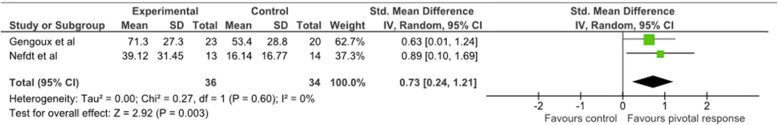
Pivotal Response Training Forest Plot

Pooling data was limited due to use of different outcome measures across the studies. Data were pooled based on three different interventions that were used by more than one study each respectively, namely DIR/Floor Time (two studies) (see Fig. 2), Parent focussed training (two studies) (see Fig. 3), and Pivotal Response Treatment (two studies) (see Fig. 4). Although there were three studies that used pivotal response treatment, as one study [39] used psychoeducation as the control intervention, this study was excluded from meta-analysis to avoid any potential contamination of data. Using a random effects model all meta-analysis showed significantly better outcomes in the intervention compared with the control group; (a) DIR, effect size: 0.98; (b) Parent focused training, effect size: 0.38; and (c) Pivotal response treatment, effect size: 0.73. There was no heterogeneity for any three treatment groups respectively (I^2^ = 0% for all three).

#### Risk of Bias

**Fig. 5:**
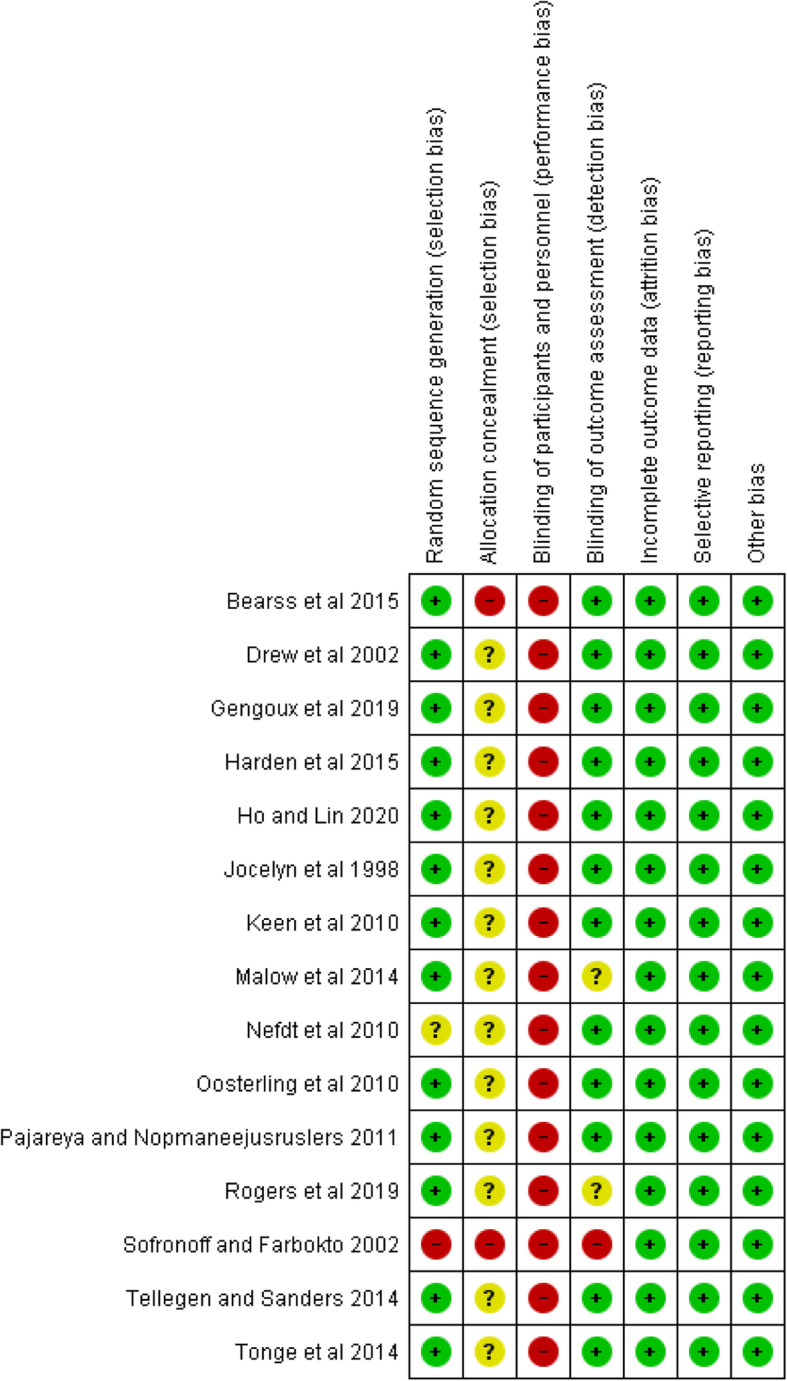
Cochrane Risk of Bias summary findings

Blinding of participants and those providing the interventions did not happen in any of the studies (see Fig. 5). This is inherent in interventions of this kind. In addition, in all the studies, it was unclear whether allocation was concealed. In Nefdt and colleagues’ study [42], it was also not clear whether there was randomisation. Finally, in Rogers and colleagues’ study [22] and Malow and colleagues’ study [24] there were uncertainties about outcome measurements concealment and bias.

## Discussion

“Parent training” is an umbrella term that refers to several disparate interventions and that the efficacy of “parent training” is dependent upon the content provided in the parent training. This systematic review included 17 papers from 15 RCTs. Two studies [24, 28] also compared outcome of delivering same intervention through two different methods.

### Design flaws in the included studies

Sixteen of the 17 papers favour interventions, although not all with a statistically significant result. However, it is well known that studies with a positive finding tend to find their way to publications more easily than the ones with a negative finding thus causing a publication bias. Although most studies have shown a positive effect of intervention on the outcomes, it is difficult to draw any definitive conclusion from this as the studies are small, and both interventions, control groups and outcomes measures are varied. This also makes it difficult to pool data for meta-analysis. Although meta-analyses showed positive treatment effects, it was only possible to pool data from two studies respectively for each of the three different specific interventions. Also, pooled data included different outcome measures. None of these are ideal for a meta-analysis and will raise question about their validity. Each group had created its own intervention which was not used by any other group for independent validation thus limiting generalisability apart from DIR, pivotal response treatment, and parent focus training each of which was used by two groups respectively. Another problem is that psychoeducation in the form of providing information through self-directed learning as opposed to a face to face training by a trainer has been used in the control arm in three studies and in the intervention arm in two studies. However, psychoeducation is used as an intervention primarily in those studies which measured parent related outcomes such as parental stress and knowledge. Therefore, psychoeducation seems an appropriate intervention in those studies. However, it is worth remembering that psychoeducation may mean many things to many people.

Another problem was that most interventions used in the included studies had multiple components. In most cases the exact details of these components were not described apart from the mention of number of sessions and time taken to deliver the intervention. As a result, it became difficult to tease out the effect of individual components of each intervention. The Risk of Bias assessment highlights the issues inherent to this kind of research; it is not possible to conceal interventions from those taking part or those delivering the assessment. Recruitment [55], as mentioned already, is another issue, so that it is difficult to mitigate against allocation bias and small sample size.

### Effect on ASD core symptoms

We found that Social Pragmatic Joint Attention Focussed Training [33, 34], Pivotal Response Training [39, 40, 42], DIR/Floor Time™ [31, 41] and Early Denver Start Model [22] are parent training interventions to improve adaptive functioning in children with ASD. Pooling data from Gengoux and colleagues [40] and Nefdt and colleagues [42] for Pivotal response training we obtained an effect size of 0.7 in favour of the training. Similarly, pooled data favoured DIR/Floor Time™ in our metanalysis (effect size: 0.98). Results were in favour of Early Denver Start Model [22] when all three sites were taken together, although not all individual centre’s results were positive. However, the evidence in support of language interventions is small. Higher DQ or IQ may predict more language acquisition. Our findings were similar to other systematic reviews who also reported a large variety of interventions and heterogeneity in outcomes [6, 9–14]. In the previous reviews, Oono and colleagues [10], Parson and colleagues [11], O’Donovan and colleagues [13], and Black and Therrien [12] looked at different kinds of parent training, delivered both face to face and remotely and found a positive trend supporting the intervention. However, a high risk of bias affected most studies as the findings were limited by low quality studies, heterogeneity of content, outcomes and outcome measurement. Children with autism are also a heterogenous group; one child’s profile of triad is very different from that of another and may depend on underlying genetic abnormality, giving rise to a specific behavioural phenotype which are beginning to be recognised [56]. Parent Training and Education need to be fine-tuned to the child’s profile to be more effective. For example, if the child is more likely to manifest repetitive behaviour than have difficulties in the area of social interactions, that is where the intervention needs to be focussed.

### Effect on associated behaviour

In terms of associated behaviour, Malow and colleagues’ [24] Sleep Education Program was able to make some changes in children’s sleep pattern. Bearss and colleagues [35] and Sofronoff and Farbotko [38] reported that the children did well after 48 weeks and 3 months respectively while there was a reduction in challenging behaviours to start with in Tellegen and Sanders [25] study. Tonge and colleagues [26] found improvement in both the treatment and the control groups. We were unable to pool data due to differences in research design used in these studies. Previous review of Posterino and colleagues [6] found a medium effect size of Parent Training on ASD children’s disruptive behaviours.

### Effect on parental outcome

Jocelyn and colleagues [29], and Keen and colleagues [28] provided education on various aspects of ASD while Bradshaw and colleagues [37] reported on the impact of active control group in Bearss and colleagues’ intervention and Iadarola and colleagues [36] on parental stress. These studies had overall shown improvement in these outcomes. However, as they were not blind, the placebo effect associated with parents getting attention from the intervention could not be ruled out. Often parents of children with ASD are under emotional stress [57], and the opportunity to discuss and receive information from a professional in itself is therapeutic for them [58].

Jocelyn and colleagues [29], and Tellegen and Sanders [25] both found using client satisfaction scales that parents were satisfied with the interventions, suggesting that it helped them to learn to see the world from their child’s perspective and approach situations in a better way. It seems that the parents value support in the early days of diagnosis [59].

All the interventions would come under Dawson Squibb and colleagues’ [60] hybrid model of Parent Education and Training (PET) defined as programmes that pass on information and/or skills to parents/carers using a range of modalities (including but not limited to didactic, role-play, discussions, video guidance) in a setting where parents/carers and trained facilitators are the direct participants. Reduction in parental stress, even in the control group is a product of parent education and training as observed in a number of studies [35, 36, 44].

### Strengths

We have used a broad criterion to capture a wide range of studies using standardised search engines and search terms. We have used PROSPERO guide to produce the design of our review. We have assessed quality of included studies using the validated Cochrane risk of bias template (see Fig. 5). We have carried out a meta-analysis using the Cochrane guideline. Our findings are in line with those of other systematic reviews in this area.

### Limitations

Our search criteria allowed for the inclusion of studies with heterogeneous methodologies and interventions, making comparison among studies and pooling of data difficult. This subsequently made it difficult to draw a definitive conclusion on the effectiveness of the interventions. Heterogeneity of interventions has been observed in several systematic reviews in this area. However, due to the absence of clear definitions for parent training, our search criteria were necessarily broad to ensure studies were not arbitrarily excluded due to wording and not identified during the literature search. Also, we have excluded conference abstracts and grey literature as we thought it would be difficult to apply the eligibility criteria for screening and assess risk of bias based on the abstracts only. We have excluded studies in which researchers provided interventions directly to children. Although this helped to avoid confounding but it also made it difficult to compare notes with previous systematic reviews. Exclusion of non-English publications may also have produced some bias. There were very little disagreements between the reviewers while screening abstracts, but the lack of interrater reliability data is a limitation.

## Conclusion

Parental training for parents of children with ASD has the potential to vastly reduce use and reliance upon medication. In addition to this, parents of children with ASD are known to experience anxiety and disempowerment in relation to their children. Training could be a valuable tool to equip parents for theirs and their children’s benefit. However, like previous systematic reviews we found a mild to moderate effect of different types of parental training on ASD symptoms of their children. Similar to other systematic reviews, we found it difficult to draw any definitive conclusion about the effectiveness and generalisability of any intervention because of the wide variation in the interventions, control groups and outcome measures used in the included studies. For training to realise its potential to minimise medication use and empower parents, a future avenue for research must be an attempt to reach consensus on how to define parent training, clarifying essential and optional features. Such a checklist will enable future systematic reviews to assess interventions in the existing evidence base and enable the inclusion of comparable interventions without the risk of unintended exclusion, facilitating informative meta-analyses. There is an urgent need for experts in various international centres to standardise a parent training intervention for children with ASD and carry out a large scale RCT to assess its clinical and economic effectiveness.

## Supplementary Information

**Additional file 1.**

## Data Availability

Not applicable to this article as no new data were created or analysed in this study.

## Abbreviations

ABC-I: Aberrant Behavior Checklist-Irritability
ADI-R: Autism Diagnostic Interview-Revised
ADOS: Autism Diagnostic Observation Scale
ASD: Autism Spectrum Disorder
CA: Chronological age
CARS: Childhood Autism Rating Scale
CGI-I: Clinical Global Impression-Improvement
CGSQ: Caregiver Strain Questionnaire
DA: Developmental age
DIR: Developmental, Individual Difference, Relationship-based
DQ: Developmental Quotient
DSM-IV-TR: Diagnostic and Statistical manual-4th edition-Text Revised
ESDM: Early Start Denver Model
FCT: Functional communication training
FEAS: Functional Emotional Assessment Scale
FEQ: Functional Emotional Questionnaires
IQ: Intelligent Quotient
ICD: International Classification of Diseases
PCSSTP: Primary Care Stepping Stones Triple P
PET: Parent Education and Training
PSI: Parenting Stress Index
PSOC: Parenting Sense of Competence
RCTs: Randomised controlled trails
TRE-ADD: Treatment Research and Education for Autism and Developmental Disorders
