# Supplementary material for: The effectiveness of parent training for children with autism spectrum disorder: a systematic review and meta-analyses
Source: BMC Psychiatry. 2020 Dec 7;20:583. doi: 10.1186/s12888-020-02973-7 (PMC7720449; doi:10.1186/s12888-020-02973-7)
Supplement: Supplementary file 1 — Additional file 1. [file 12888_2020_2973_MOESM1_ESM.docx]

**Supplementary file 1:** Search terms (Deb et al).

**Medline:** psychoeducation, parents, children, child, autism, autistic, autistic spectrum disorder, ASD, autis*, autistic disorder, autism disorder, intellectual disability, intellectual disorder, learning disorders, attention deficit hyperactivity disorder, adhd, attention disorder deficit with hyperactivity, hyperkinetic disorder, teaching, training, education, support, parents.

**EMBASE:** psychoeducation, parents, children, child, autism, autistic, autistic spectrum disorder, ASD, autis*, autistic disorder, autism disorder, intellectual disability, intellectual disorder, learning disorders, intellectual impairment, attention deficit hyperactivity disorder, adhd, attention disorder deficit with hyperactivity, hyperkinetic disorder. teaching, training, education, support, parents.

**PsycInfo**: psychoeducation, parents, children, child, autism, autistic, autistic spectrum disorder, ASD, autis*, autistic disorder, autism disorder, intellectual disability, intellectual disorder, learning disorder, Intellectual development disorder, attention deficit hyperactivity disorder, adhd, attention disorder deficit with hyperactivity, hyperkinetic disorder. teaching, training, education, support, parents.

**CINAHL:** psychoeducation, parents, children, child, autism, autistic, autistic spectrum disorder, ASD, autis*, autistic disorder, autism disorder, intellectual disability, intellectual disorder, "LEARNING DISORDERS", attention deficit hyperactivity disorder, adhd, attention disorder deficit with hyperactivity, hyperkinetic disorder. teaching, training, education, support, parents.
